# Individual parental conversations with non-birthing parents

**DOI:** 10.1017/S1463423620000286

**Published:** 2020-07-30

**Authors:** Margaretha Larsson, Irene Eriksson, Karin Johansson, Anna-Karin Stigsson, Rebecka Svahn, Johanna Wetterström, Marie Wilhsson

**Affiliations:** 1 School of Health Sciences, University of Skövde, Skövde, Sweden; 2 Primary Health Care in Skövde, Sweden; 3 Home Health Care in Skövde, Sweden

**Keywords:** child healthcare, father, non-birthing parent, nursing, primary healthcare, qualitative content analysis

## Abstract

**Aim:**

The aim of this study was to describe Child Health Service (CHS) nurses’ experiences with conducting individual parental conversations (IPCs) with non-birthing parents.

**Background:**

CHS nurses in Sweden mainly focus on monitoring a child’s physical and mental development and the mothers’ health in order to support their parenthood. The assignment of the CHS includes identifying dysfunctional social relationships in a family and strengthening responsive parenting. An imbalance arises within the family when someone in the family suffers from illness, which could have a negative effect on the whole family’s health and well-being.

**Methods:**

An inductive, descriptive qualitative study design was used to describe and to gain an understanding of the CHS nurses’ experiences. Data were collected in 13 interviews, and a qualitative content analysis was performed.

**Findings:**

The analysis of interviews with CHS nurses resulted in two main categories, each with three subcategories. The main categories are: working for equality and applying a family focus, and dealing with challenges in the developing assignment. The IPCs stimulate the CHS nurses to work for more equality and to apply a family focus, which can be a way of strengthening the families’ health and the children’s upbringing. Developing the CHS nurses’ assignment can be a challenge that appears to entail positive outcomes for CHS nurses, while also generating the need for CHS nurses to receive supervision to find ways to improve their approach and practice.

## Background

Child healthcare is an important arena for providing support to new parents. Access to high-quality family planning services is fundamental for realizing the rights and well-being of women as well as men and girls and boys (WHO/RHR and CCP, 2018). The Child Health Service (CHS) program in Sweden is based on the Conventions on the Rights of the Child (UNICEF, 1989) with a child perspective and the child’s best in focus. CHS in Sweden offers free support and general healthcare for children under school age as well as their families. The goal is to promote child health by providing health examinations, guidance and vaccinations for all children, as well as parental support. The CHS nurses meet the child and parents the first time when the child is newborn, often in a home visit and after that at a CHS center. The CHS is staffed by professionals, such as physicians and CHS nurses and with access to a psychologist, a dietician, a social worker and a speech therapist (Tell, 2019). The CHS assignment includes identifying unhealthy social relationships in the family and strengthening responsive parenting (Swedish National Board of Health, 2014). Interaction between the children and their parents is to be promoted, and the CHS nurse is to monitor and observe the parents’ mental health. CHS nurses have, both historically and more recently, mainly focused on monitoring the child’s physical and mental development and the mothers’ health to support their parenthood (Wells et al., 2017). This has occurred in spite of research showing that the involvement of both parents in the child’s upbringing promotes the health and development of the child (Hallberg et al., 2005; Singh, 2017; Barboza et al., 2018; Zhang et al., 2019). Furthermore, a study from four The Organisation for Economic Co-operation and Development (OECD) countries shows that an overwhelming majority of fathers took some time off work to be involved in the daily care of their child (Huerta et al., 2013).

The transition to parenthood is a stressful period for most parents both as individuals and as couples, with variations in the parents’ mental health and linked to the children’s long-term health. It is important to support parents during the transition to parenthood using different approaches and being sensitive to the unique needs of both the mothers and their non-birthing parents (Gilmer et al., 2016). The national program of the CHS is based on family-focused care, which means that each member of the family together forms a unit called the family (Benzein et al., 2008). Several international studies show positive effects on the health of the whole family when support is not only given to the mothers, but also to the fathers in the transition to being a parent (Sarkadi et al., 2008; Plantin et al., 2011; Coleman and Ahmann, 2016; Bäckström et al., 2018).

An imbalance arises within the family when someone in the family suffers from illness, which could have a negative effect on the whole family’s health and well-being (Corlett and Twycross, 2006; Coleman and Ahmann, 2016). Parents’ illnesses are described as stressful for children, making them vulnerable to emotional and behavioral problems (Chen, 2017), which can have a negative effect on the children’s cognitive and emotional development (Edhborg et al., 2003). Risk factors for parents’ illness could, according to Massoudi et al. (2016), be relationship problems, a lack of support from the father, a low level of education, negative life events and previous illness. Approximately 10–20% of mothers around the world suffer from postpartum depression (Beck et al., 2006), and 8–15% of mothers in Sweden suffer from postpartum depression 6–8 weeks after childbirth (SBU, 2014). The validated screening tool, the Edinburgh Postnatal Depression Scale (EPDS), is used in the CHS to identify postpartum depression when the child is eight weeks old. The EPDS measures the mothers’ experiences of joy, guilt, concern, fear, sadness and thoughts of harming themselves (Cox et al., 1987). International studies show that 8–10% of fathers report ill-health such as postpartum depression (Paulson and Bazemore, 2010; Paulson et al., 2016), and 6–10% of the fathers in Sweden demonstrate symptoms of depression three to six months after the birth of the child (Kerstis et al., 2013; Massoudi et al., 2016). Nurses at the CHS can use the EPDS if they suspect mental illness and for identifying it in the father, although this is not included in the child health program (Massoudi et al., 2011; Massoudi et al., 2013). Massoudi et al. (2013) show that the EPDS identifies more distress than just depression in new fathers. They maintain that EPDS is a valid instrument for screening for probable major depression, but it is questionable whether it should be used to screen for minor depression. According to Paulson and Bazemore (2010), an individual dialogue with the father between three and six months after the birth could promote health. The importance of including the father in the CHS program through extended father conversations in order to identify illness in the family has been emphasized in several studies (Plantin et al., 2011; Liu et al., 2016; Massoudi et al., 2016).

Coyne et al. (2013) and Harvey and Ahmann (2014) describe the importance of nurses listening to parents without any assessment of what parents say in order to strengthen the relationship between the nurse and the parent. Coyne et al. (2013) maintain it is important to create space for conversation between nurses and families in order to build confidence and trust in the family. It is also important to create a trusting relationship between the nurse and the father (Fägerskiöld, 2006). The possibility for both parents to be involved in the child’s care on equal terms is increased when the father feels included, and it will also be more natural for the father to participate in the care of the child (Fägerskiöld, 2006; Massoudi et al., 2011; Bäckström et al., 2017; Barboza et al., 2018), and Hrybanova et al. (2019) have shown that fathers had positive experiences of support when CHS nurses provided practical information and stimulated them to be involved in care of their children.

## Aim

The aim of this study was to describe CHS nurses’ experiences with conducting individual parental conversation (IPC) with non-birthing parents.

## Method

### Design

An inductive, descriptive qualitative study design was used to describe and to gain a greater understanding of the CHS nurses’ experiences with conducting IPC with the non-birthing parent. Data were collected in face-to-face interviews, and a qualitative content analysis, as described by Graneheim and Lundman (2004) and Graneheim et al. (2017), was performed.

### The intervention

**Figure 1.**
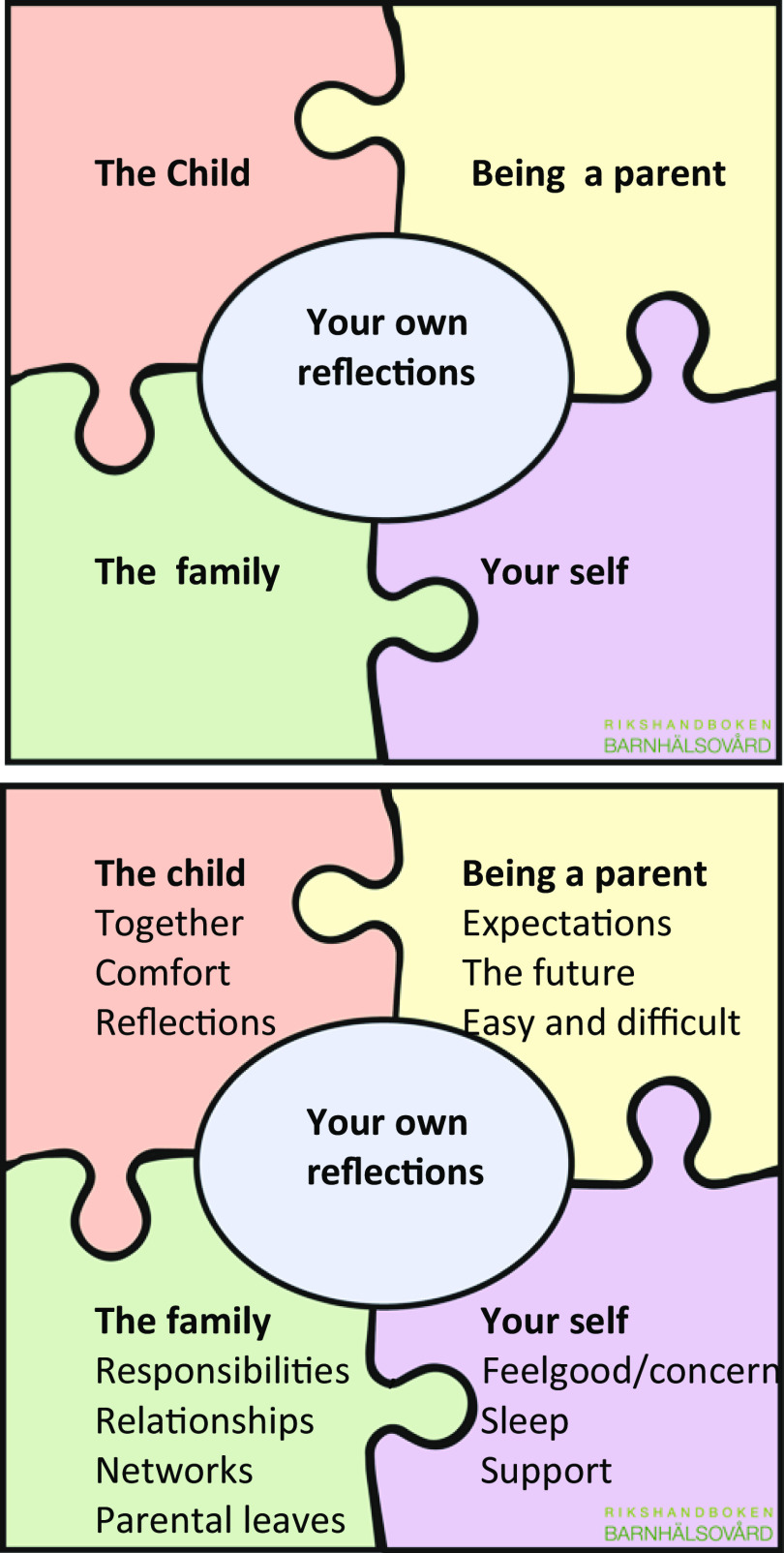
The four-piece jigsaw puzzle

The intervention consisted of non-birthing parents (could be a man or a person with the same sex) being offered an IPC with a CHS nurse. The overall aim of the intervention was to promote the non-birthing parents’ health and to offer support to non-birthing parents with a newborn child. The CHS nurses used a material consisting of a four-piece jigsaw puzzle and a question guide in the IPC. The material was based on a previous Swedish intervention, which was developed and tested in three regional health authorities in eastern and southern Sweden (Bergström et al. 2017; Bergström, 2019; Stahl et al., 2019). The four-piece jigsaw puzzle and question guide concerned four areas; the child, being a parent, the family and health. The pieces of the puzzle had the name of the area on one side and the name of the area and some support concepts on the other (Figure 1). The CHS nurses received a one-day training session for using the material, which was supplemented with additional opportunities for follow-up questions and discussion. The intervention was offered to a total of 32 CHS centers, of which three were interested in performing IPC with non-birthing parents in their CHS.

### Participants

The study took place in three municipalities in urban areas in western Sweden. All the CHS nurses in the three CHS centers (*n* = 13) who were working with IPC were asked to participate, and all of them agreed after receiving verbal and written information. All the participants were women and Registered Nurses with specialist education in primary healthcare or pediatric care. Their work experience varied between four and 35 years (median = 8). All of them had experience using the IPC with non-birthing parents as a father and some of them as a same-sex parent.

#### Data collection

Data were collected in qualitative face-to-face interviews with the aim of understanding the participants’ experience using the IPC with non-birthing parents. The interviews were semi-structured, and the interview guide was constructed collaboratively by all authors. All the interviews started with the same question: ‘How did you experience having an individual conversation with the non-birthing parent?’ The questions covered their experience with the material they used and how they used it, what new knowledge they had gained when using this material and how they would like to continue working with this new knowledge. The interviews were carried out by the authors (KJ, A-KS, RS & JW) in January 2018 in a secluded setting at the participants’ workplace. The interviews were audio-recorded, lasted for approximately 20 min and were transcribed verbatim.

#### Data analysis

Qualitative content analysis, influenced by Graneheim and Lundman (2004), was used. The text was initially read through several times by all the authors in order to become familiar with the text and to reflect upon the content. Meaning units that reflected the purpose of the study were identified and condensed in the second stage of the analysis, and then, the essential content was abstracted and labeled with a code. The codes related to the content of the meaning units then emerged. The codes were sorted into two main categories and six subcategories, based on similarities and differences in the final fourth stage of the analysis, which was an iterative process back and forth across the stages and not a linear process. All the authors were involved in all stages of the analysis, and the findings were discussed and reflected upon. In order to secure trustworthiness, the interviews were analyzed separately, compared and discussed by all authors to reach consensus about the final categories (Graneheim et al., 2017).

#### Ethical considerations

This study followed the ethical principles of autonomy, beneficence, non-maleficence and justice in the Declaration of Helsinki (World Medical Association, 2017), and ethical approval was not needed for this type of study in accordance with Swedish legislation. Permission to conduct the study was given by the three directors of the CHS centers. Oral and written information was given to the participants, and all of them gave informed oral and written consent to participate in the study. The information provided concerned the voluntary nature of and confidentiality in participation, and the right to withdraw from the study at any time without explanation.

## Result

The analysis resulted in two main categories, each with three subcategories. The main categories were identified as follows; ‘Working for equality and applying a family focus’ and ‘Dealing with challenges in the developing assignment.’ The main categories with their subcategories are described in detail below (see also Table 1). Quotations are used to illustrate the findings.

**Table 1.** The main categories and subcategories

Main category	Subcategory
Working for equality and applying a family focus	Creating a relationship Strengthening the family Promoting participation and equality
Dealing with challenges in the expanding assignment	Creating conditions for conversation Bridging barriers to participation in IPC Developing with the assignment

### Working for equality and applying a family focus

CHS nurses experience IPC with non-birthing parents as a way of working for equality and applying a family focus, which can be a way of strengthening both of the parents in their parenthood. In order to be able to support non-birthing parents, it was essential to create a relationship. Their hope was that this could lead to positive effects for the children’s upbringing.

#### Creating a relationship

CHS nurses experienced IPC as essential for creating a relationship with the non-birthing parents. The nurses stated that most non-birthing parents received the offer of an IPC in a positive way and that they appreciated the interest in them as a person and of being in focus. This was also the case for the non-birthing parents who previously had children. The CHS nurses reported that the IPC strengthened their relationships with the non-birthing parents, which was enriching and facilitated their work. This could lead to an increased sense of trust that could help the non-birthing parent to be able to contact the CHS nurse on other occasions.

…if one has built a bridge to a father/partner then it is much easier for them to make contact…it feels as if we are opening several doors both for them and for us

The first home visit with a newborn child was an important opportunity for inviting the non-birthing parents to the IPC. CHS nurses emphasized that relationships that start during a home visit form the foundation for a different understanding of the families and the family members than if they had only met the non-birthing parent at the CHS’ centers. Offering individual conversations to both the mothers and the non-birthing parents was experienced as being natural and positive.

…and it feels good to have something to offer to the partner … that he also gets something, we know that they can also feel bad occasionally

There could be difficulties talking about the IPC when the non-birthing parent was not present at the first home visit, and the information about it was only conveyed by the mothers instead of directly to the non-birthing parents. CHS nurses reported that the mothers could say that their non-birthing parent did not need an IPC, but on the occasions when the non-birthing parent did still participate in an IPC, the CHS nurses felt that the conversation was appreciated, and a relationship was created.

#### Strengthening the family

CHS nurses felt they had the opportunity to strengthen the family when they had a greater family focus in their work by offering both parents individual conversations. The parents’ narrative could differ depending on whether it was the mother or the non-birthing parent who spoke. It resulted in them gaining new and increased knowledge about the non-birthing parents’ well-being and role in the family, which increased the CHS nurses’ understanding of the families’ situation and provided a better overall picture of the families.

….you learn something from each encounter, you always get a little something, I remember having a father here with his third child and it had been quite a struggle, but I should not had that type of conversation before with that father and the conversation with him became a little eye-opener for me, a bit more understanding for how difficult it was, so it gave me a completely different picture …

The CHS nurses experienced that the conversations with the non-birthing parents could entail the latter gaining an understanding of the importance of being a parent, which can, in turn, lead to a stronger relationship with and an increased responsibility for the child. The CHS nurses worked to confirm the non-birthing parent’s role in the family and had hopes that the conversation could lead to the non-birthing parent opening up more at home and thereby strengthening the relationship between the parents.

…it is just that they get a different relationship with the child, and it is also good for their relationship together, because if one talks more about feelings and what one feels like then one might do it at home as well. It becomes sort of more permitted, the whole family is very important

The non-birthing parents have spoken of both positive and negative aspects in the IPC, such as their concerns about married life after the birth of the child and the parents’ relationship with each other. The CHS nurses described having raised questions with the aim of visualizing factors that can affect the non-birthing parents’ health and discuss what help they may desire and what was possible, for example, to make appointments with other healthcare providers and parent counselors.

…I met one partner who did not feel well while we were speaking, and he was able to break it down into separate parts that he could see and was also able to work out what the cause was

The CHS nurses reported that both the mothers and non-birthing parents appreciated that the non-birthing parent had the opportunity to reflect on their health, parenthood and family life.

#### Promoting participation and equality

The CHS nurses described that having an understanding of gender equality and participation should be self-evident for the CHS and that it is equally important to strengthen the non-birthing parent in the parental role as the mother. They reported that they consciously thought about including the non-birthing parents in their work at the CHS in order to create participation and promote equality. Their experience was that the non-birthing parents appreciated that both parents were involved. The IPC was seen as a natural part of the development in their work for equal parenthood as well as in the society. The CHS nurses felt that the non-birthing parents participated more in the children’s appointments at the CHS health center after the IPC had been conducted.

…that they also come at the time for the second appointment. And that feels good then … that they feel more involved

The CHS nurses hoped that the parents would feel that it did not matter which of them came to the appointments at the CHS health center. They maintained that increased participation promoted equality in parenting and could lead to attention being paid to the importance of the family and where the aim was for all the family to feel well.

…I generally feel very favorable about this, it is something that gives something to the families and to us as well

Inviting non-birthing parents to an IPC in connection with the child’s first vaccination was a good opportunity, because both parents often come to that appointment.

### Dealing with challenges in the expanding assignment

CHS nurses felt that they dealt with the challenges in their expanding assignment using the intervention material in a personal way and being flexible in order to bridge barriers that could facilitate the non-birthing parents’ participation in the IPC. Furthermore, they felt they were successful in their professional development in relation to their assignment.

#### Creating conditions for conversation

The CHS nurses’ experience was that the jigsaw puzzle and the guide with the questions created suitable conditions for the IPC with non-birthing parents. The puzzle and the questions provided a structure for and support in creating a natural dialogue. The CHS nurses reported that the pieces of the puzzle were easy to understand and were specific when providing a visual guide during the conversation. Furthermore, they served as a framework for the conversation and could be used to guide the conversation if necessary. The guide with questions was used differently by the CHS nurses; some memorized the questions while others had the guide with them as support. Having the pieces of the puzzle in front of them facilitated the non-birthing parents to gain an overall picture of what the conversation would include according to the CHS nurses, even if the pieces of the puzzle were talked about separately, and the areas that were important for the non-birthing parent were discussed in more detail.

It is very specific, I think, because there are a few instructions about what can be discussed and that is good for me and good for the father or partner, because they then know something about what may come up while at the same time it is not too regulated, so that they can bring up what they want to talk about

The CHS nurses adapted the pieces of the puzzle to their personal way of working as well as to the non-birthing parents’ expectations, needs and personality. The IPC varied depending on which probing questions were asked to each individual non-birthing parent. The pieces of the puzzle were perceived as being flexible and were also used for summarizing what the conversation had focused on and what they had agreed upon.

I think they are good when you want to sum up, when you try to round off the conversation and tie things up that we have now talked about this matter and that matter

#### Bridging barriers to participation in IPC

The CHS nurses experienced that they needed to bridge over barriers that were related to time and culture as well as traditions in order to conduct the IPC with the non-birthing parents. Barriers related to time concerned difficulties in finding times that were suitable for the non-birthing parents. The CHS nurses meant that when the non-birthing parents returned to their everyday routines, they did not consider taking time off from their work in order to participate in the IPC, but wished to have times that were early or late in the day. Another difficulty arose when the CHS nurses only worked part time, which, thus, meant there was a limit to the number of times available to offer to the non-birthing parent.

Being as I do not work full time, the most difficult thing is to find a good time, because we try to book a time that suits the partners, so that they can come here, and then, they most often want to come quite late in the afternoon, so that they can take a little time off from work …

The CHS nurses tried to find solutions that created possibilities for the non-birthing parents to attend the IPC. They dealt with this by extending the period of time for the IPC to be conducted. A problem arose when the non-birthing parent was unable to attend the IPC at the time of the checkups when their child was three to four months old, which was held at the CHS centers.

Barriers related to culture concerned conversations with non-birthing parents from other cultures and when interpreters were required. The CHS nurses felt insufficient when the non-birthing parent did not speak Swedish or English and experienced that it was difficult to create a natural conversation when having to talk through an interpreter. They found it difficult to know exactly what the interpreter said, and they felt that the questions were not always understood. They interpreted this as being related to the non-birthing parent’s culture, that it was not as natural to talk about one’s health or that the non-birthing parents did not understand the importance of the conversation. The CHS nurses thought that the non-birthing parents from other cultures generally said that everything was very good or tended to decline to come to the ICP.

The only occasions that I thought were difficult or tricky were when the parents born abroad… when one has to have an interpreter… they come from completely different circumstances and when I ask them then everything is most often very good

Barriers related to traditions were experienced when the non-birthing parent questioned the IPC and the CHS nurses’ routines. The CHS nurses felt that the expectations and norms in society affected the non-birthing parents’ attitude to the IPC. They said that the non-birthing parents could act as though they had been picked out specially.

… they have not heard that their friends go to such things… not their siblings either, why should they go? They think it is something special for them

The CHS nurses felt that they needed to have a leaflet about the purpose of the IPC that they could give to the non-birthing parents during the first home visit.

#### Developing with the assignment

The CHS nurses reported that they felt that they had developed personally as they carried out their assignment. Conducting the IPC successfully made them feel competent, which entailed that the conversation became easier and more natural to perform. They said that there were significant differences between the IPC and other visits made to the CHS centers. The child was always present when the mother came to the CHS, but even when the child was not present during the IPC, he/she was to be in focus. To be able to perform an IPC, the CHC nurses ask the mothers to leave the room, which was seen as a challenge when they take the children with them. The CHS nurses reported difficulties when they had to readjust from having an appointment with a young child present to having a conversation with the non-birthing parent.

…you suddenly have to sit there face to face without the child being there. It is really the child that we work with, but also with the parents of course. If they do not feel well, then the child does not feel well

The CHS nurses experienced feelings of inadequacy in terms of a lack of knowledge and resources. The introduction of the IPC meant that they had more tasks to perform but with the same conditions. They spoke about feeling as though they were counselors during the IPC but lacked knowledge, for example, when the non-birthing parents talked of their concerns about the family’s economy. Feelings of inadequacy also arose when there were not sufficient resources to take care of mental ill-health in primary care, which meant that they could not refer the non-birthing parents, but were alone in having responsibility for supporting the non-birthing parents’ needs. This generated reflections about the meaning of implementing the IPC if they were not able to provide adequate help.

I have had to work a lot with my own feelings. It was really difficult at the start, I have not wanted to work with psychology or be any sort of psychologist, but have wanted to work with children and parents

The CHS nurses’ experience was that none of the non-birthing parents had left the IPC with any form of distress. They perceived that the non-birthing parents were grateful for the possibility to talk about their health and life situation by the way they showed their appreciation and gratitude when leaving the CHS.

… they shook hands and thanked us, a mother has probably never done that

## Discussion

The CHS nurses experienced that the IPC was a task that promoted the non-birthing parents’ participation and equality as well as strengthening the family focus, which enriched and facilitated their work in promoting family health and supporting the children’s upbringing. The IPC provided an important opportunity to establish a relationship with the non-birthing parents, which entailed supporting the non-birthing parents’ understanding of the importance of being a parent, which could lead to a stronger relationship with and increased responsibility for the child. The CHS nurses strove to confirm the non-birthing parent’s role in the family and had hopes that the conversation could lead the non-birthing parent to be more open at home and thus strengthen the relationship between the parents. Earlier research has showed that it is important to include both the fathers and mothers in the care of the child (Massoudi et al., 2011), and when the non-birthing parent feels that they are included and their individual needs are taken into account, their opportunities to become involved in the new role as a parent increase (Bäckström et al., 2017). Parry et al. (2019) show that for some men, a male-only conversation offers a space to feel comfortable to open up to discuss sensitive topics. The National Board of Health and Welfare (2009) emphasizes that healthcare is to be provided to everyone on equal terms. The Swedish CHS program was developed in 2019 to include an IPC with the non-birthing parent (Bergström, 2019).

The result shows that a wide range of aspects that the non-birthing parents needed to talk about were discussed in the IPC. When the CHS nurses discovered factors that affected the non-birthing parents’ health, they strove to help them to discern them and see what they could depend on; then, they discussed what assistance that they desired and was possible. This is important, since research shows that many fathers experience illness such as depressive symptoms during the postnatal period (Goodman, 2004; Paulson and Bazemore, 2010; Cameron et al., 2016). Fathers with postnatal depression engage less in their interaction with their infants (Sethna et al., 2017). Structured methods for assessing mental illness within the family are necessary, especially as men do not generally seek help spontaneously for mental health concerns (Cheung and Dewa, 2007; Boman and Walker, 2010; Seidler et al., 2016). This also highlights the importance of gender equality, and the CHS nurses’ approach should be developed, so that both parents are seen as equal in terms of support for their health. A greater number of fathers are actively engaging in parenting in several countries, which also increases the impact of the father’s parental behavior on child outcomes (Sarkadi et al., 2008).

The IPC was experienced as being problematic by the CHS nurses when the child was not present but still in focus, which forced them to focus on the non-birthing parents’ perspective. Furthermore, the IPC could also arouse feelings of inadequacy related to a lack of knowledge and a lack of resources and services to which they could refer the non-birthing parents. Corlett and Twycross (2006) maintain that those who work with families are expected to have professional knowledge for promoting and supporting the whole family in their daily lives and for cooperating with other professions. They found it difficult to cooperate with external services when few resources were available, thus forcing the CHS nurses to rely on themselves. The material the CHS nurses had at their disposal consisted of four pieces of puzzle and a question guide as an aid, which they experienced as creating the conditions for the IPC. They felt that the pieces of puzzle were easy to understand and were explicit as they provided visual guidance, and that the pieces could also serve as a framework for the conversation and guide the conversation if required. Having a material that provided a structure that they could adapt to their own personal way of working as well as the non-birthing parents’ expectations, needs and personality was important. Stahl et al. (2019) maintain that healthcare professionals such as CHS nurses need proper guidance and education when expanding their duties and responsibilities to include non-birthing parents in individual conversations. The CHS nurses in this study received training for one day, which was supplemented with additional opportunities for follow-up questions and discussions during the period they performed the IPC. By having additional opportunities to discuss and be trained in how to support the non-birthing parents, the CHS nurses can be strengthened, and to be able to support both the mother and the non-birthing parent in their parenthood, the CHS nurses need competence in talking to and involving both parents at the CHS health centers whether the child is present or not. Having access to such a material, as described here, appears to have helped to structure the conversations.

The result shows that most non-birthing parents received the offer for an IPC in a positive way, even those who had children previously, and the CHS nurses felt that the non-birthing parents appreciated the interest shown in them as a person and to be in focus. However, some of the non-birthing parents questioned their invitation to an IPC and the CHS nurses spoke of the non-birthing parents acting as though they had been specifically selected, which the CHS nurses felt was related to societal expectations and norms that affected the non-birthing parents’ attitude to the IPC. This situation needs to be considered from the perspectives of both the professionals and the non-birthing parents. Research shows that CHS providers feel ambivalent toward engaging fathers in the care of children (Allport et al., 2019). Massoudi et al. (2011) report that CHS nurses can have an ambivalent attitude toward the caring capacity of fathers compared to mothers. Another aspect is that CHS nurses traditionally turn to the mothers when meeting with parents (Fägerskiöld, 2006), and in spite of research in Sweden showing that child health nurses perceived a greater gender equality in 2014 than in 2004, mothers still receive the majority of the parenting support (Wells et al., 2017). Providing supervision for CHS nurses can be a way of supporting them to make changes. Bell (2018) maintains that it is important to learn from failure and to have opportunities to discuss one’s own practice in order to enhance skills in family nursing to achieve well-founded practice. Supervision of nursing supervisors using an ethical approach focusing on patient-related situations has led to shared knowledge that strengthened the supervised profession (Berggren and Severinsson, 2003), and to supporting the development of the supervisee’s job identity, competence, skills and ethics (Severinsson, 2001). Driscoll et al. (2019) maintain that supervision helps nurses by providing evidence of their continuing professional development. Further education based on new research in the subject can develop the CHS nurses’ knowledge about the positive associations between the father’s involvement and engagement with the child’s cognitive and physical health outcomes (Aronen and Arajarvi, 2000; Ramchandani et al., 2013). To better correspond to the non-birthing parents’ perspective, a written folder describing the purpose of the IPC could be handed over when they receive the invitation for the IPC, so the non-birthing parents know that this is something for all new parents.

The CHS nurses felt inadequate when performing an IPC with non-birthing parents who did not speak Swedish or English. Using an interpreter made it difficult to create a natural conversation. Berlin et al. (2006) have claimed that primary child healthcare nurses lack cultural competence, which has an effect on their ability to meet the needs and expectations of this group of children and parents. Furthermore, it can inhibit them from gaining trust when they attempt to provide adequate healthcare. The CHS nurses described that the non-birthing parents from other cultures usually talked of everything being very good or tended to decline to participate in the IPC, which can be a result of the nurses lacking cultural competence. It has been shown in a study of an intervention among CHS nurses focusing on cultural competence that this aspect has improved, which can, in turn, improve the quality of CHS and, thus, can reduce the risk for health-care disparities among children (Berlin et al. 2010).

### Limitations

This study covered a specific region of Sweden, and the fact that the study is based on a sample of female participants may be a limitation of the study. Some of the interviews were short, which can be considered as a limitation; however, the interviews generated rich data that provided a deeper understanding of the CHS nurses’ experiences of conducting the IPC with the non-birthing parent as a way of supporting them in their parenthood and working family-focused. The intervention IPC with non-birthing parents was a new mission for the CHS nurses, and research that validates the intervention, use of jigsaw, is missing, which can be a limitation. The procedures and methods were presented as thoroughly as possible in an attempt to prove credibility and to make it possible for the reader to consider and understand the logic of the findings. Moreover, quotations were used to show that the findings were grounded in the interview texts in order to assure conformability. The researchers are the instrument in qualitative studies, which implies that the development of self-awareness is of importance, as well as the researchers’ competence, thoroughness and integrity in assessing the findings (Polit and Beck, 2018). The researchers had all of these factors in mind throughout the process. Qualitative results can never be universal, but knowledge can still be transferred to contexts other than the original one (Dahlberg et al. 2008). However, the findings from this study could be transferable to CHS nurses in other countries meeting non-birthing parents at CHS.

### Conclusion

The IPC with non-birthing parents helps the CHS nurses to work more equally and to apply a family focus, which can be a way of strengthening family health and the children’s upbringing. Expanding the CHS nurses’ assignment can be a challenge, but it appears to mostly entail positive outcomes for the CHS nurse and the non-birthing parents they meet. This study indicates that CHS nurses need support and further education to have the pre-requisites to provide the IPC with a high quality of healthcare. Performing the IPC concurs with one of the aims of CHS, which is to work with family-focused care. Furthermore, the practical support provided by the material was useful for conducting the IPC, but a folder is needed that describes the IPC and which can be handed over to the non-birthing parents at the time of the invitation.
